# Phosphorus Allocation to Leaves of Beech Saplings Reacts to Soil Phosphorus Availability

**DOI:** 10.3389/fpls.2019.00744

**Published:** 2019-06-06

**Authors:** Sonia Meller, Emmanuel Frossard, Jörg Luster

**Affiliations:** ^1^Forest Soils and Biogeochemistry, Swiss Federal Institute for Forest, Snow and Landscape Research WSL, Birmensdorf, Switzerland; ^2^Institute of Agricultural Sciences, ETH Zürich, Zurich, Switzerland

**Keywords:** acclimation, beech, *Fagus sylvatica*, forest health, phenotypic plasticity, phosphorus allocation, phosphorus nutritional status, soil phosphorus availability

## Abstract

Decreasing phosphorus (P) concentrations in leaves of beech (*Fagus sylvatica* L.) across Europe raise the question about the implications for forest health. Considering the distribution of beech forests on soils encompassing a broad range of nutrient availability, we hypothesized that this tree species exhibits high phenotypic plasticity allowing it to alter mass, and nutrient allocation in response to local nutrient availability. To test this, we grew two groups of 12–15 year old beech saplings originating from sites with high and low soil P availability for 2 years in mineral soil from their own site and in soil from the other site. After two growing seasons, P concentrations in leaves and stem, as well as mass allocation to leaves and fine roots were affected by both soil and plant origin. By contrast, relative P allocation to leaves and fine roots, as well as P concentrations in fine roots, were determined almost entirely by the experimental soil. Independent of the P nutritional status defined as average concentration of P in the whole plant, which still clearly reflected the soil conditions at the site of plant origin, relative P allocation to leaves was a particularly good indicator of P availability in the experimental soil. Furthermore, a high plasticity of this plant trait was indicated by a large difference between plants growing in the two experimental soils. This suggests a strong ability of beech to alter resource allocation in response to specific soil conditions.

## Introduction

Forests dominated by beech (*Fagus sylvatica* L.) cover a large part of Europe (ca 14–15 Mha) from southern Norway to southern Italy and from northern Spain to northwest Turkey (Brunet et al., 2010; Durrant et al., 2016). Throughout this area, beech populations occur on a wide variety of soils realizing a broad ecological niche in terms of soil chemical properties including pH (3.2–7.3), base saturation (3–99%), and plant-available phosphorus (P) pools in the mineral topsoil (11–1287 mol P m^–2^ 10 cm^–1^) (Leuschner et al., 2006; Batjes, 2011; Yang et al., 2013).

Analysis of data from forest monitoring plots (ICP forest level II) across Europe between 1991 and 2010 revealed a significant decline in P concentrations and an increase of N/P ratios in beech leaves on the majority of plots (Jonard et al., 2015; Talkner et al., 2015) confirming findings of earlier regional studies (Flückiger and Braun, 1998; Duquesnay et al., 2000; Jonard et al., 2009; Braun et al., 2010). These changes in leaf nutrient status have been attributed to continuing high nitrogen (N) deposition and increasing atmospheric carbon dioxide concentrations. Both accelerated tree growth due to high N and carbon (C) input and negative effects of elevated N on fine root biomass, mycorrhization, and litter mineralization appear to have created a higher need for other nutrients which cannot be met by the supply from the soil (Kjøller et al., 2012; Peñuelas et al., 2013; Talkner et al., 2015). On 40% of the level II plots the average leaf P concentrations indicated P deficiency (Jonard et al., 2015) and average N/P ratios on 60% of the plots were higher than “good for harmonious nutrition” (Talkner et al., 2015). The currently widely accepted critical values for nutrient concentrations and ratios in leaves (Mellert and Göttlein, 2012) are based on a large number of studies comparing leaf values with deficiency symptoms, growth or reaction to fertilization. However, results from surveys and experiments on the relation between P concentrations in plant tissues and soil P availability are partially conflicting. While concentrations of P in leaves and fine roots of mature trees across different forest sites were not well related to measures of P availability in the soil (Talkner et al., 2015; Lang et al., 2017), this relation was stronger for saplings from two acid forest sites and considering different plant compartments including leaves, stem, fine and coarse roots (Yang et al., 2016; Zavišić and Polle, 2018; Zavišić et al., 2018). Furthermore, Zavišić et al. (2018) observed a strong increase of P concentrations in all compartments upon fertilization. One reason for the conflicting results could be that often only total P concentrations in soil are available which not sufficiently reflect this elements bioavailability. Another explanation could be the strong seasonal and site dependent variability of P concentrations in plant tissues (Yang et al., 2016; Netzer et al., 2017; Zavišić and Polle, 2018). This points to the importance of seasonal dynamics of nutrient uptake and plant internal nutrient allocation.

However, surprisingly little is known on how beech adapts its internal P allocation to different P availability in soil, and thus to what degree P concentrations in leaves directly reflect soil P availability or the ability of the tree to regulate its P content on the cellular and the whole-plant level (Marschner, 1996). On the cellular level, P homeostasis can be maintained by adjusting the flux of P into and out of the vacuole (Lee et al., 1990; Lee and Ratcliffe, 1992; Mimura et al., 1996). On the whole plant level, P redistribution is regulated via vascular tissues, and it follows seasonal patterns employing storage in and recycling from various organs including senescing leaves, roots, and stem (Raghothama, 1999; Lin et al., 2009, 2014; Brant and Chen, 2015; Netzer et al., 2017; Zavišić and Polle, 2018; Zavišić et al., 2018). The seasonally varying relations between soil P availability and internal P cycling have been shown for P concentrations in various plant compartments comprising leaves, fine roots, coarse roots and stem (Netzer et al., 2017; Zavišić and Polle, 2018), for P concentrations in xylem and phloem (Yang et al., 2016; Netzer et al., 2017), and for P resorption from older and senescent leaves (Hofmann et al., 2016; Netzer et al., 2017). Furthermore, growth rates of adult beech not always reflected soil P availability (Netzer et al., 2017) highlighting the potential role of high-efficiency internal cycling in trees growing on soils with low P availability. Alteration of internal nutrient cycling can be part of the adaptive response (phenotypic plasticity) of plants to external factors. For many plants, phenotypic plasticity was shown to comprise rapid trait alterations during a lifetime of an organism (Sultan, 2004; Hodge, 2009), including morphological, anatomical, physiological, reproductive, and developmental traits. Considering the long time scales of migration and natural selection, phenotypic plasticity is the most important asset of tree populations to cope with the ongoing environmental changes (Matesanz et al., 2010; Nicotra et al., 2010; Vitasse et al., 2010). The phenotypic plasticity of beech in response to drought or increased temperature has been well studied, comprising plant traits such as phenology (e.g., Kramer, 1995; Vitasse et al., 2009), biomass and root morphology (Curt et al., 2005; Weemstra et al., 2016), leaf anatomy (Stojnić et al., 2015a, 2015b), stem anatomy (Stojnić et al., 2013; Diaconu et al., 2016), mass allocation, and growth rate (Rolo et al., 2015).

By contrast, little is known about the adaptive response of beech to changes in P availability. Our objective was therefore to identify the respective most responsive traits of this tree species. Considering the important role the plant nutritional status plays in governing nutrient acquisition (Marschner, 1996), we performed experiments with beech saplings adapted to grow in soil with either high or low soil P availability, and assessed their response to contrasting soil conditions. We hypothesized that the response of a beech sapling with a given P nutritional status, as defined by its site of origin, to higher or lower soil P availability in terms of biomass and P allocation to different plant compartments is mainly driven by the plant striving to increase a low foliar P concentration as fast as possible or to maintain a high foliar P concentration as long as possible.

## Materials and Methods

### Plant and Soil Materials

Plant and soil materials were collected on the core research sites of the priority program 1685 “Ecosystem nutrition” of the German and Swiss National Science Foundations^1^ in Unterlüss (LUE, Lower Saxony, Germany) with low P availability in the soil, and Bad Brückenau (BBR, northern Bavaria, Germany) with high P availability in the soil. The sites were chosen because they both sustain mature mono-specific beech stands but differ profoundly in soil P stocks and cycling. For details on the sites refer to Lang et al. (2017).

Saplings of beech (*F. sylvatica*) of similar size (approx. 45 cm in height and approx. 8 mm base diameter) were gently dug out at the sites during their dormancy period in December 2014, and stored at 4°C with their roots embedded in soil from their own site until planting.

Soil materials were collected from the Bh horizon in LUE and the Bv horizon in BBR, air-dried at 15°C, sieved to 4 mm, and homogenized. Plant residues were removed from the soils. Basic physical and chemical properties are listed in Table 1 and were obtained as follows. Soil texture was determined using the pipette method (Gee and Bauder, 1986). Soil pH was measured in a 1:2 slurry in 0.01 M CaCl_2_ or water after 30 Min. equilibration. Organic C (C_org_) and total N (N_tot_) contents of ground soil samples were measured using an elemental analyzer (NC 2500, CE Instruments Ltd, Hindley Green, Wigan, United Kingdom). Exchangeable cations (Al_ex_, Ca_ex_, Fe_ex_, K_ex_, Mg_ex_, Mn_ex_, and Zn_ex_) were extracted with 1M NH_4_Cl for 1 h at 20°C and a soil:extractant ratio of 1:10. The filtered extracts were analyzed for total elemental concentrations by inductively coupled plasma optical emission spectrometry (ICP-OES; Optima 7300 DV; Perkin Elmer, Waltham, MA, United States). Sequential P extraction was performed according to Hedley et al. (1982) as modified by Tiessen and Moir (2006). In Table 1, resin exchangeable inorganic P (P_resin_), the sum of inorganic P (P_inorg_) in various extracts (0.5 M NaHCO_3_, 0.1 M NaOH before and after sonication, 1 M HCl, concentrated HCl) and the sum of organic P (P_org_) in the NaHCO_3_ and NaOH extracts are shown. The soil from BBR exhibited much higher concentrations of both inorganic and organic extractable P than the LUE soil, but most importantly also resin exchangeable inorganic P, a measure of inorganic P in soil solution, and loosely sorbed to soil particles and thus of available P (Tamburini et al., 2012), was much higher in the BBR soil.

**TABLE 1:** Physical and chemical properties of homogenized material from the Bv soil horizon at Bad Brückenau (BBR) and the Bh soil horizon at Unterlüss (LUE) used in the experiment; this includes grain size fractions, pH in two different extractants, exchangeable metal cations (M_ex_), organic C (C_org_), total N (N_tot_), and the following P fractions obtained by sequential extraction: resin exchangeable inorganic P (P_resin_), sum of inorganic P (extractable P_inorg_) and organic P (extractable P_org_) in various extracts (for details see text); all concentrations are given per mass dry soil; shown are means ± standard deviations of two technical replicates, except for sum parameters and element ratios.

		**LUE**	**BBR**
Sand	(g kg^–1^)	811 ± 3	287 ± 14
Clay	(g kg^–1^)	43 ± 4	253 ± 14
pH in H_2_O/ 0.01 M CaCl_2_		3.99 ± 0.01/ 3.31 ± 0.01	4.76 ± 0.04/ 3.99 ± 0.01
Al_ex_	(mmol_c_ kg^–1^)	19.7 ± 0.2	40.6 ± 0.2
Ca_ex_	(mmol_c_ kg^–1^)	0.56 ± 0.01	2.13 ± 0.04
Fe_ex_	(mmol_c_ kg^–1^)	1.35 ± 0.02	0.04 ± 0.006
K_ex_	(mmol_c_ kg^–1^)	0.49 ± 0.02	0.56 ± 0.04
Mg_ex_	(mmol_c_ kg^–1^)	0.33 ± 0.003	0.62 ± 0.01
Mn_ex_	(mmol_c_ kg^–1^)	0.10 ± 0.003	0.79 ± 0.01
Zn_ex_	(mmol_c_ kg^–1^)	0.02 ± 0.001	0.03 ± 0.001
C_org_	(g kg^–1^)	18.5 ± 0.04	41.9 ± 1.0
N_tot_	(g kg^–1^)	0.75 ± 0.01	3.22 ± 0.01
P_resin_	(mg kg^–1^)	0.44 ± 0.04	5.5 ± 1.3
Extractable P_inorg_	(mg kg^–1^)	29	911
Extractable P_org_	(mg kg^–1^)	89	1256
C_org_/N_tot_	(g g^–1^)	24.7	13.0
C_org_/P_org_	(g g^–1^)	208	33
N_tot_/P_org_	(g g^–1^)	8.4	2.6

### Experimental Setup

In April 2015, rhizoboxes were set up with beech saplings planted either in the soil from their site of origin or in the contrasting soil from the other site. In a completely randomized design, each treatment was replicated seven times. The rhizoboxes had inner dimensions of 60 cm × 25 cm × 1.5 cm. They consisted of PVC walls and a removable transparent lid made of polymethyl methacrylate. The soil was filled in at a bulk density of 1.2 kg/dm^3^. After 1 week of soil conditioning under irrigation as described below, the saplings were planted. The roots of the saplings were washed with tap water to remove sticking soil, and approximately 2 cm of tap root were cut to stimulate new root formation. For each tree, the front plate of one rhizobox was opened, the roots pressed into the soil, and the front plate was closed again. At this time point, saplings possessed up to 10 cm long tap roots of 0.5–1.5 cm diameter but almost no fine roots, which presumably had died off during the storage. Rhizoboxes with trees were placed in a greenhouse with temperature control (22 ± 2°C during the day/18 ± 2°C at night), natural light and shading from the direct sun. Since shading with movable blinds was the only means for active cooling, at some days in summer temperatures higher than 22°C occurred for short periods. The soil was kept dark, and to stimulate the formation of a quasi-planar root system along the transparent lid, the rhizoboxes were inclined at an angle of about 30°. Soil water potential in the rhizoboxes was kept at approximately -8 kPa by using irrigation tubes (“Rhizon irrigators,” Rhizosphere research products, Wageningen, Netherlands) providing P-free artificial rain solution based on the composition of natural precipitation [2.1 μM K_2_SO_4_, 3.7 μM Na_2_SO_4_, 3.0 μM CaCl_2_, 4.4 μM CaSO_4_, 1.9 μM MgCl_2_, 26.4 μM NH_4_NO_3_, 2.0 μM Ca(NO_3_)_2_; Holzmann et al., 2016]. During summer, additional periodic irrigation from the top was needed to compensate for high evapotranspiration. At the end of the first growing season (end of September 2015), the rhizoboxes were placed outside of the greenhouse, but protected by a roof, to induce dormancy. In November 2015, they were moved to a dark cold room at 4°C and periodically irrigated with artificial rain from the top. End of March 2016, after the last frost, the rhizoboxes were moved first to the protected area outside of the greenhouse, and in May, after appearance of the first leaves, back into the greenhouse with temperature control set to the same conditions as in the year before.

### Plant Harvest and Analyses

During the first growing season in August 2015, when plants reached the phenological stage of fully developed leaves in both soils (Yang et al., 2016), five fully expanded leaves per plant were collected. Senescent leaves were collected at the end of the season after natural leaf abscission (December 2015) into nets spread around the plants. In August 2016 of the second growing season, the whole plants were harvested. At that time point, six saplings each from BBR growing in soil from BBR and LUE, 7 saplings from LUE growing in soil from BBR, and 3 saplings from LUE growing in soil from LUE had survived. The plants were divided into leaves, stem, coarse roots, and fine roots (diameter ≤ 2 mm).

The following analyses were performed on fresh tissue samples. The age of the saplings at final harvesting was determined by staining thin sections of the stem and subsequent tree-ring analysis (Gärtner and Schweingruber, 2013). According to this, the saplings from BBR were 11.7 ± 2.7 years old, and those from LUE 14.7 ± 1.6 years old. Subsamples of fully developed leaves were used to measure the trichloroacetic acid (TCA)-soluble P fraction (also called metabolic P; Wilcox et al., 2000). Approximately 200 mg of fresh leaves were frozen in liquid N_2_ in a 15 ml reagent tube and crushed to a fine powder using metal beads and vortexing. Powdered leaves were extracted for 1 h at 4°C with 4 ml of 0.3 M TCA on a shaker. Extracts were filtered at 0.45 μm using glass fiber GF/F filters (Whatman International Ltd.). Inorganic P concentrations in TCA extracts were measured colorimetrically using malachite green (Van Veldhoven and Mannaerts, 1987). Bark and wood exudates were collected with the EDTA (Ethylene diamine tetra acetate) technique (Rennenberg et al., 1996; Gessler et al., 1998; Yang et al., 2016). Briefly, approximately 2 cm of the basal stem was collected fresh and separated into bark and wood. These parts were washed with deionized water and incubated in 2 ml of a solution 10 mM in Na_2_EDTA (pH 7) and 15 μM in chloramphenicol for 5 h at room temperature. Then, inorganic P in the incubation solution was measured colorimetrically as described above.

Plant parts not used for the analyses described above, were oven dried at 60°C for 48 h (leaves and fine roots) or 72 h (stems and coarse roots), weighed, and ground to fine powder using a ball mill (Retsch MM400 Mixer Mill, Retsch GmbH, Retsch-Allee 1–5, 42781 Haan, Germany) with receptacle and balls made of agate. Total carbon and nitrogen contents of the ground material were measured by combustion using an elemental analyzer (NC 2500, CE Instruments Ltd, Hindley Green, Wigan, United Kingdom). The contents of total P were determined by ICP-OES (Optima 7300 DV; Perkin Elmer, Waltham, MA, United States) of digests obtained with a solution 8.3 M in HNO_3_ and 0.6M in HF using a microwave digestion unit (MW ultraCLAV, MLS, Milestone Inc., Shelton, CT, United States).

### Mass and P Allocation Parameters

Biomass allocation to a specific plant compartment was calculated as mass fraction (g) of the compartment in percent of the total dry mass of the whole plant (g) (Poorter and Sack, 2012). Phosphorus allocation to a specific plant compartment was calculated as the mass fraction of P (g) in the plant compartment in percent of total plant P (g).

Resorption efficiency (RE) of nutrient elements X (X = P, N) from senescent leaves collected in December 15 was estimated by Eq. 1.

(1) $$\text{RE}(\text{X})=\left(1-\left(\frac{(1-0.21)\;\times \;\mathrm{Xs}}{\mathrm{Xf}}\right)\right)\;\times \;100$$

Here Xs and Xf stand for nutrient concentrations in senescent and full season leaves, respectively. As no specific data on mass loss during senescence for beech was available, we used the average value of mass loss (21%) based on a multiple species analysis by Van Heerwaarden et al. (2003). A similar average mass loss of 21.6% was reported for “deciduous angiosperms” by Vergutz et al. (2012).

Considering the crucial role that xylem plays for P recycling in beech (Netzer et al., 2017), the P sink strengths of leaves (S_Leaves_), and fine roots (S_fine roots_) were calculated as total P concentration in leaves and fine roots, respectively, divided by concentrations of inorganic P in wood exudates.

### Plasticity Indices

We adapted the concept of plasticity indices (Valladares et al., 2006) to quantify the change of a given plant trait during the response of a beech sapling with a given P nutritional status to changes in soil P availability. For more on rationalizing the following equations see the respective subsection of “Discussion.”

In a first step, we calculated the average potential span for a given trait (ΔTr) as the arithmetic mean of pairwise differences between trait values of the m and n replicates, respectively, within the two treatments with beech saplings from BBR (TR_BBRinBBR_) and LUE (TR_LUEinLUE_) growing in soil from their own site using Eq. 2.

(2) $$\Delta \mathrm{Tr}=\frac{|\sum_{i=1..n,j=1..m}\left({\mathrm{Tr}}_{\mathrm{BBR in BBR}}(i)-{\mathrm{Tr}}_{\mathrm{LUE in LUE}}(j))\right|}{m*n}$$

Only traits with significant differences between the mean trait values for the two treatments were taken into account.

In a second step, we calculated average plasticity indices for the response of saplings from LUE to soil with high P availability (PL_LUEplant_) and for the response of saplings from BBR to soil with low P availability (PL_BBRplant_). The plasiticity indices were calculated as the arithmetic mean of pairwise differences between trait values of the m and n replicates, respectively, within two treatments with beech saplings from a given site growing in soil from their own site and in soil from the other site, divided by the trait span (Eqs. 3 and 4).

(3) $${\mathrm{PL}}_{\mathrm{LUE plant}}=\left(\frac{|\sum_{i=1..n,j=1..m}\left({\mathrm{Tr}}_{\mathrm{LUE in BBR}}(i)-{\mathrm{Tr}}_{\mathrm{LUE in LUE}}(j))\right|}{m*n}\right)/\Delta \mathrm{Tr}$$

(4) $${\mathrm{PL}}_{\mathrm{BBR plant}}=\left(\frac{|\sum_{i=1..n,j=1..m}\left({\mathrm{Tr}}_{\mathrm{BBR in BBR}}(i)-{\mathrm{Tr}}_{\mathrm{LUE in LUE}}(j))\right|}{m*n}\right)/\Delta \mathrm{Tr}$$

### Statistical Analysis

Using analysis of variance (ANOVA), we assessed to what extent current soil (the soil in which the beech saplings were growing during the experiment) on one hand, and plant origin (forest site where the beech saplings were collected) on the other hand, influenced the measured plant traits in the first and second growing season. All ANOVA analyses were performed in R, version 3.1.2 (R Core Team, 2014; **RRID:SCR_001905**) with marginal type II test (Anova, package: “car”) in order to account for unequal group sizes. Prior to ANOVA, data was subjected to Levene’s test (leveneTest, package: “stats”) for homogeneity of variance inside the groups and to the Shapiro-Wilk normality test (shapiro.test, package: “stats”) for normality of residuals. Statistical significances indicated with letters in figures and tables are the result of a one-factorial ANOVA (with treatment as explanatory variable) with a Tukey *post hoc* test (HSD.test, package: “agricolae”). Average plasticity indices for plants from BBR and LUE were compared using multiple t-tests. Each pair was analyzed individually, without assuming a consistent standard deviation, using GraphPad Prism 7.02 Software. The standard error of the mean for the plasticity indices was computed for the true sample size using Gaussian error propagation and assumption of no error in ΔTr.

## Results

### Foliar Element Concentrations and Nutrient Resorption in the First Growing Season

**FIGURE 1:**
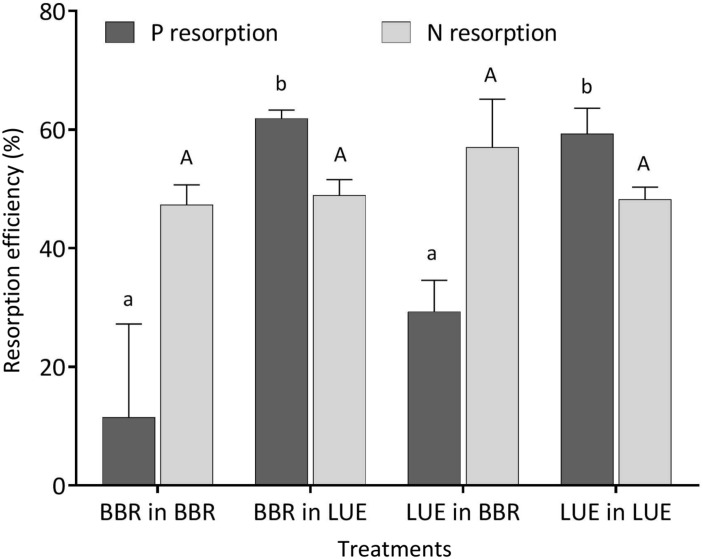
Resorption efficiency for P (black bars) and N (gray bars) of beech (*Fagus sylvatica* L.) saplings in the first growing season of the experiment; saplings originated from the sites Bad Brückenau (BBR) with high soil P availability and Unterlüss (LUE) with low soil P availability, and were grown in material from the Bv horizon at BBR or from the Bh horizon at LUE; data represent mean values ± SE for the different treatments; different letters indicate significant differences between means according to the Tukey *post hoc* test (lowercase letters, P resorption; uppercase letters, N resorption).

During the first growing season, both total and metabolic P concentrations in leaves were slightly higher for beech saplings originating from BBR, the site with high soil P availability, than for those from LUE, the site with low soil P availability (Table 2). According to ANOVA, both concentrations were significantly affected only by the factor “plant origin” (Table 3). The first measured reaction of the saplings to the soil they were growing in during the experiment was a higher P resorption from senescent leaves for all plants growing in LUE soil than for those growing in BBR soil, irrespective of plant origin (Figure 1). ANOVA revealed “current soil” as sole significant factor (Table 3). On the other hand, N resorption from senescent leaves did not differ among the treatments and was not affected neither by plant origin nor by current soil conditions (Figure 1 and Table 3).

**TABLE 2:** Concentrations of P (total P, P_tot_; metabolic P, P_metabolic_) and total N (N_tot_) in full season and senescent leaves of beech (*Fagus sylvatica* L.) saplings, measured in the first growing season of the experiment; saplings originated from the sites Bad Brückenau (BBR) with high soil P availability and Unterlüss (LUE) with low soil P availability, and were grown in material from the Bv horizon at BBR or from the Bh horizon at LUE; data represent mean concentrations per unit mass dry weight ± SE; different letters indicate significant differences between means according to the Tukey *post hoc* test.

			**BBR in BBR**	**BBR in LUE**	**LUE in BBR**	**LUE in LUE**
Full season	P_tot_	(mg g^–1^)	1.23 ± 0.09 ab	1.42 ± 0.14 a	0.94 ± 0.06 b	1.04 ± 0.13 ab
	P_metabolic_	(mg g^–1^)	0.35 ± 0.07 ab	0.38 ± 0.05 a	0.12 ± 0.01 c	0.17 ± 0.05 bc
	N_tot_	(mg g^–1^)	21.5 ± 0.9 a	21.0 ± 0.9 a	23.3 ± 0.4 a	24.2 ± 1.5 a
Senescent	P_tot_	(mg g^–1^)	1.35 ± 0.23 a	0.68 ± 0.05 b	0.83 ± 0.05 b	0.54 ± 0.13 b
	N_tot_	(mg g^–1^)	14.2 ± 0.7 a	13.5 ± 0.7 a	15.3 ± 0.4 a	16.0 ± 1.7 a

**TABLE 3:** Analysis of variance for different traits of beech (*Fagus sylvatica* L.) saplings as determined in the first growing season of a rhizobox experiment; traits include concentrations of P (total P, P_tot_; metabolic P, P_metabolic_) and total N (N_tot_) in full season and senescent leaves as well resorption efficiency for P and N; saplings originated from the sites Bad Brückenau (BBR) with high soil P availability and Unterlüss (LUE) with low soil P availability (factor plant origin), and were grown in material from the Bv horizon at BBR or from the Bh horizon at LUE (factor current soil); shown are *F* values for the factors and their interactions; statistical significance is indicated as ^∗∗∗^*P* < 0.001, ^∗∗^*P* < 0.01, ^*^P < 0.05, ns *P* > 0.05.

		**Source of variation**		
		**Current soil**	**Plant origin**	**Current soil × plant origin**
Full season leaves	P_tot_	1.97 ns	9.93^∗∗^	0.15 ns
	P_metabolic_	1.71 ns	33.0^∗∗∗^	0.30 ns
	N_tot_	1.15 ns	18.6^∗∗^	0.05 ns
Senescent leaves	P_tot_	24.0^∗∗∗^	10.8^∗∗^	0.67 ns
	N_tot_	0.01 ns	4.17 ns	0.50 ns
Resorption efficiency	P_tot_	26.4^∗∗∗^	0.03 ns	0.29 ns
	N_tot_	0.27 ns	0.82 ns	0.86 ns

### Phosphorus Concentrations in Different Plant Compartments in the Second Growing Season

In the second growing season, total P concentrations in stem and coarse roots, and average concentrations in the whole plant still reflected the soil P availability at the site of plant origin with higher values for the saplings from BBR than for those from LUE (Table 4). Plant origin dominated as factor in the ANOVA (Table 5). By contrast, the P concentrations in fine roots were significantly affected mainly by the factor current soil (Table 5) and were higher for saplings growing in the BBR soil (Table 4). Total and metabolic P concentrations in leaves as well as inorganic P concentrations in bark and wood exudates were similarly affected by both plant origin and current soil (Table 5), which led to higher values for the saplings from BBR growing in mineral soil from their own site than for all other treatments (Table 4).

**TABLE 4:** Concentrations of P (total P, P_tot_; metabolic P, P_metabolic_; inorganic P in bark exudates, P_Bark_Exudates_; inorganic P in wood exudates, P_wood_exudates_) in and biomass of different compartments of beech (*Fagus sylvatica* L.) saplings, measured in the second growing season of a rhizobox experiment; saplings originated from the sites Bad Brückenau (BBR) with high soil P availability and Unterlüss (LUE) with low soil P availability, and were grown in material from the Bv horizon at BBR or from the Bh horizon at LUE; data represent mean concentrations per unit mass dry weight (concentrations) or g dry weight (biomass) ± SE; different letters indicate significant differences between means according to the Tukey *post hoc* test.

			**BBR in BBR**	**BBR in LUE**	**LUE in BBR**	**LUE in LUE**
Leaves (full season)	P_tot_	(mg g^–1^)	1.53 ± 0.12 a	0.93 ± 0.09 b	0.92 ± 0.07 b	0.72 ± 0.27 b
	P_metabolic_	(mg g^–1^)	0.67 ± 0.07 a	0.18 ± 0.02 b	0.19 ± 0.04 b	0.20 ± 0.11 b
	Biomass	(g)	1.62 ± 0.13 a	1.03 ± 0.16 b	1.18 ± 0.09 ab	0.37 ± 0.16 c
Stem	P_tot_	(mg g^–1^)	0.81 ± 0.03 a	0.66 ± 0.04 b	0.36 ± 0.03 c	0.28 ± 0.07 c
	P_Bark_exudates_	(mg g^–1^)	0.134 ± 0.007 a	0.092 ± 0.006 b	0.071 ± 0.006 bc	0.052 ± 0.006 c
	P_Wood_exudates_	(mg g^–1^)	0.016 ± 0.001 a	0.009 ± 0.001 b	0.007 ± 0.0004 b	0.004 ± 0.001 c
	Biomass	(g)	4.8 ± 0.4 a	4.0 ± 0.6 a	5.8 ± 1.0 a	4.5 ± 0.4 a
Coarse roots	P_tot_	(mg g^–1^)	0.76 ± 0.07 a	0.66 ± 0.11 a	0.31 ± 0.02 b	0.22 ± 0.05 b
	Biomass	(g)	8.8 ± 0.5 a	8.1 ± 0.4 a	8.2 ± 0.3 a	6.3 ± 0.1 b
Fine roots	P_tot_	(mg g^–1^)	0.95 ± 0.04 a	0.66 ± 0.02 b	0.88 ± 0.07 a	0.48 ± 0.04 b
	Biomass	(g)	2.57 ± 0.35 a	1.49 ± 0.40 ab	1.86 ± 0.23 ab	0.62 ± 0.18 b
Whole plant	P_tot_	(mg g^–1^)	0.88 ± 0.05 a	0.69 ± 0.07 b	0.43 ± 0.03 c	0.27 ± 0.05 c
	Biomass	(g)	17.7 ± 1.0 a	14.6 ± 1.5 ab	17.2 ± 1.3 a	11.7 ± 0.5 b

**TABLE 5:** Analysis of variance for different traits of beech (*Fagus sylvatica* L.) saplings as determined in the second growing season of a rhizobox experiment; traits include, for different plant compartments, concentrations of P (total P, P_tot_; metabolic P, P_metabolic_; inorganic P in bark exudates, P_Bark_Exudates_; inorganic P in wood exudates, P_wood_exudates_), biomass, sink strength, and relative allocation of total P and biomass; saplings originated from the sites Bad Brückenau (BBR) with high soil P availability and Unterlüss (LUE) with low soil P availability (factor plant origin), and were grown in material from the Bv horizon at BBR or from the Bh horizon at LUE (factor current soil); shown are F values for the factors and their interactions; statistical significance is indicated as ^∗∗∗^*P* < 0.001, ^∗∗^*P* < 0.01, ^*^P < 0.05, ns *P* > 0.05.

		**Source of variation for P concentrations, biomass, or sink strength**			**Source of variation for P or mass allocation**		
		**Current soil**	**Plant origin**	**Current soil × plant origin**	**Current soil**	**Plant origin**	**Current soil × plant origin**
Leaves	P_tot_	13.8^∗∗^	12.6^∗∗^	3.08 ns	41.8^∗∗∗^	2.73 ns	0.24 ns
(Full season)	P_metabolic_	17.6^∗∗^	20.0^∗∗∗^	8.92^∗∗^			
	Biomass	29.0^∗∗∗^	17.8^∗∗^	0.80 ns	33.6^∗∗∗^	35.3^∗∗∗^	2.39 ns
	Sink strength	1.24 ns	10.1^∗∗^	2.07 ns			
Stem	P_tot_	10.6^∗∗^	126^∗∗∗^	1.04 ns	3.68 ns	5.78^∗^	2.83 ns
	P_Bark_exudates_	23.8^∗∗∗^	67.6^∗∗∗^	2.68 ns			
	P_Wood_exudates_	43.8^∗∗∗^	77.7^∗∗∗^	4.38 ns			
	Biomass	3.05 ns	1.94 ns	0.02 ns	0.36 ns	19.2^∗∗∗^	1.06 ns
Coarse roots	P_tot_	4.01 ns	54.6^∗∗∗^	0.49 ns	7.00^∗^	6.63^∗^	0.00 ns
	Biomass	10.9^∗∗^	8.04^∗^	2.79 ns	6.77^∗^	0.57 ns	0.12 ns
Fine roots	P_tot_	45.8^∗∗∗^	5.38^∗^	1.16 ns	10.1^∗∗^	2.34 ns	1.95 ns
	Biomass	13.5^∗∗^	6.25^∗^	0.07 ns	12.4^∗∗^	7.78^∗^	0.12 ns
	Sink strength	2.69 ns	34.4^∗∗∗^	0.09 ns			
Whole plant	P_tot_	11.9^∗∗^	72.4^∗∗∗^	0.09 ns			
	Biomass	11.0^∗∗^	1.36 ns	0.91 ns			

**FIGURE 2:**
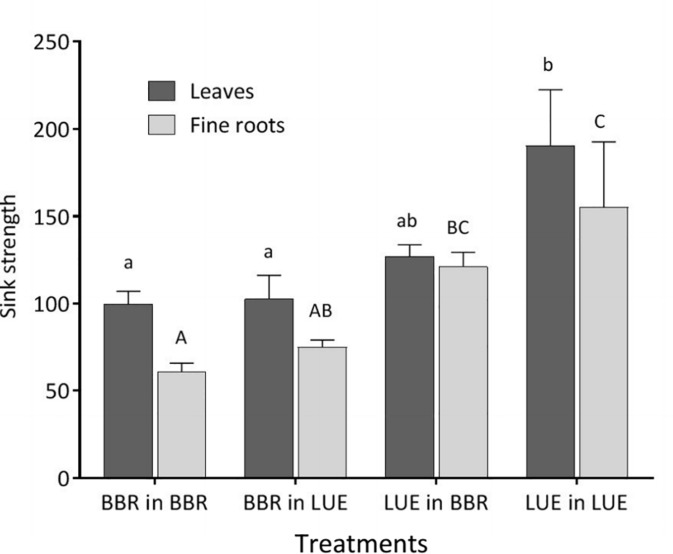
Sink strength of leaves (black bars) and fine roots (gray bars) of beech (*Fagus sylvatica* L.) saplings in the second growing season of the experiment; saplings originated from the sites Bad Brückenau (BBR) with high soil P availability and Unterlüss (LUE) with low soil P availability, and were grown in material from the Bv horizon at BBR or from the Bh horizon at LUE; data represent mean values ± SE for the different treatments; different letters indicate significant differences between means according to the Tukey *post hoc* test (lowercase letters, leaves; uppercase letters, fine roots).

The sink strengths of leaves and roots were still clearly dominated by the factor plant origin (Table 5) and were higher for the saplings from LUE (Figure 2).

### Biomass and P Allocation to Leaves and Roots in the Second Growing Season

Total plant biomass and biomass of leaves and roots were mainly and significantly affected by the factor current soil (Table 5), with the smallest values for saplings from LUE growing in soil from their own site (Table 4). By contrast, stem biomass did not differ among the treatments.

**FIGURE 3:**
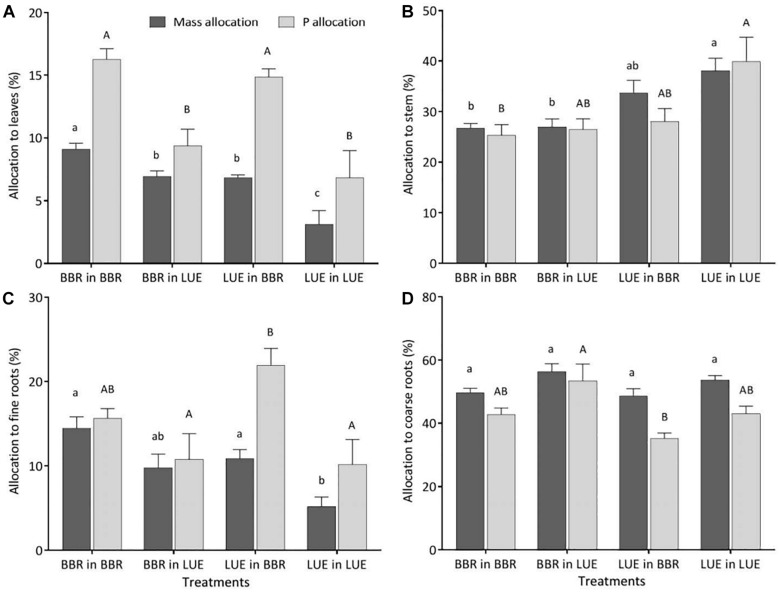
Relative allocation of biomass (black bars) and P (gray bars) to different compartments of beech (*Fagus sylvatica* L.) saplings (**A**: leaves; **B**: stem; **C**: fine roots; **D**: coarse roots) in the second growing season of the experiment; saplings originated from the sites Bad Brückenau (BBR) with high soil P availability and Unterlüss (LUE) with low soil P availability, and were grown in material from the Bv horizon at BBR or from the Bh horizon at LUE; data represent mean values ± SE for the different treatments; different letters indicate significant differences between means according to the Tukey *post hoc* test (lowercase letters, mass allocation; uppercase letters, P allocation).

Irrespective of the treatment, the largest percentage of biomass and P was allocated to coarse roots (Figure 3D). Of all measured plant traits, relative allocation of P to leaves most clearly reflected the factor current soil (Table 5) with significantly higher values for beech saplings growing in the BBR soil, irrespective of their site of origin (Figure 3A). Also, P allocation to fine roots exhibited a significant influence of current soil (Table 5), with values tending to be higher for saplings growing in BBR soil (Figure 3C). Noteworthy was the clearly highest value of all treatments for saplings from LUE growing in soil from BBR. On the other hand, allocation of biomass to the stem was still mainly determined by plant origin (Table 5) with a tendency to higher values for saplings from LUE (Figure 3B). A significant but similarly strong influence of both current soil and plant origin was detected for the allocation of biomass to leaves and fine roots (Table 5), with the highest and lowest values for the saplings from BBR and LUE, respectively, growing in their own soil (Figures 3A,C).

### Plasticity Indices

**FIGURE 4:**
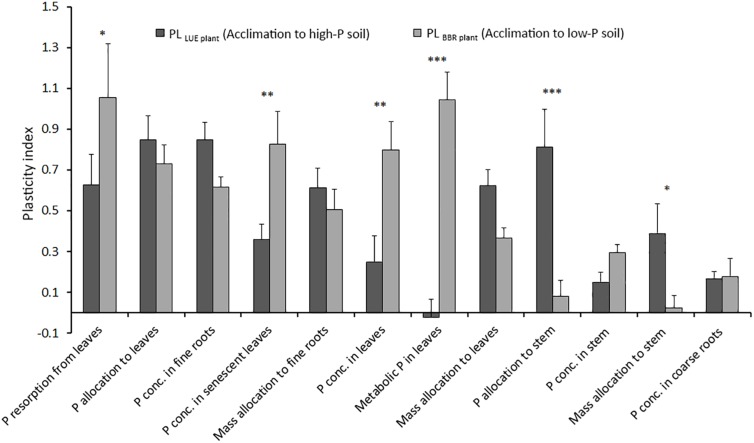
Average plasticity indices for the response of beech (*Fagus sylvatica* L.) saplings from the site Unterlüss (LUE) with low P nutritional status to mineral soil from Bad Brückenau (BBR) with high P availability (black bars) and for saplings from the site BBR with high P nutritional status to mineral soil from LUE with low P availability (gray bars); data represent mean values ± SE of all data pairs; statistical significance is indicated as ^∗∗∗^*P* < 0.001, ^∗∗^*P* < 0.01, ^*^P < 0.05.

Figure 4 shows the plasticity indices for beech saplings from LUE and BBR considering all plant traits for which the precondition of a significant difference between the two treatments with the saplings growing in soil from their own site was fulfilled. Total and metabolic P concentrations in full season leaves, total P concentrations in senescent leaves, and P resorption were more plastic for beech saplings from BBR responding to soil with low P availability. On the other hand, P and mass allocation to stem were more reactive for beech saplings from LUE acclimating to soil with high P availability. For all other traits, plasticity was similar for beech saplings from both origins, and thus independent on the direction of acclimation. However, values differed strongly with particularly high indices for P allocation to leaves and P concentration in fine roots and particularly low indices for P concentrations in stem and coarse roots.

## Discussion

Our experimental model systems employed beech saplings from forest sites strongly differing in soil P availability in terms of both measures of available P such as P_resin_ and total P stocks (Lang et al., 2017), BBR with high, and LUE with low P availability. At the end of the experiment, the saplings from the two sites still exhibited a significantly different P nutritional status in terms of average P concentration in the whole plant. Using material from mineral soil horizons from the two sites as model soils, while providing homogeneous material for the experimental replicates, possibly changed soil P availability compared to the natural situation, in particular in the case of the LUE site, where 50% of the P stock is stored in the organic surface layer (Lang et al., 2017). Nevertheless, in the second growing season total P concentrations in all plant compartments where similar to beech saplings collected at the same sites in the same year as our saplings, but were excavated together with an undisturbed soil core, and further grown this way for one season in the greenhouse (Zavišić et al., 2018).

The results of our experiment thus allow to discuss the influence of P nutritional status on the plant internal allocation of P and biomass for beech saplings growing in soil with different P availability. We consider the treatments with saplings growing in the soil from their own site to represent the situation of plants adapted to a given soil situation. We furthermore assume that the results from the treatments with saplings growing in the soil from the other site provide clues on the plastic response of beech that allows it to acclimate to changes in soil nutrient conditions.

### Foliar P Concentrations in Beech Depend on Both Soil P Availability and P Nutritional Status of the Plant

As pointed out in the introduction, P concentrations in tissues of trees can strongly vary with the season (Eschrich et al., 1988; Netzer et al., 2017; Zavišić and Polle, 2018) which indicates site dependent dynamics of nutrient uptake and plant internal nutrient allocation. Some studies even suggest that P utilization in beech can be decoupled from P uptake, i.e., the growth of young leaves may strongly depend on the transport of nutrients stored in the previous season in organs such as stem and coarse roots, but also from older leaves during a growing season (Schachtman et al., 1998; Güsewell, 2004; Zavišić et al., 2018). This could well explain our observations, that during the first growing season, total and metabolic P in leaves reflected the P availability of the soil at the site of plant origin rather than the soil in which the beech saplings were growing during the experiment. The results of the second growing season point to combined effects of current soil and plant origin. On one hand, the low P concentrations in leaves and roots of the beech saplings growing in the LUE soil, irrespective of their site of origin and thus their P nutritional status, were not surprising. On the other hand, the leaves of LUE saplings growing in BBR soil exhibited equally low P concentrations, despite high P uptake as indicated by relatively high P concentrations in fine roots. Taken together, and considering in addition the equally high mass allocation to leaves for LUE trees growing in BBR soil and for BBR trees growing in LUE soil, these results point to the ability of beech to deal with different soil P availability optimally at a given P nutritional status of the plant by internal allocation of P and mass. It suggests a priority to alleviate the primary light limitation by producing as much photosynthetic organs as possible, while vacuolar P concentrations are kept low when soil P availability and/or the P nutritional status of the whole plant is low. The latter has been shown to be a successful mechanism in other plants (Lee et al., 1990; Lee and Ratcliffe, 1992; Mimura, 1995; Mimura et al., 1996). Comparing the leaf P concentrations in the second growing season with the threshold values for young beech trees published by Göttlein (2015) suggests the following. While a P concentration within the normal range (1.1–2.1 mg/g DW) indicates both a high P nutritional status of the plant and high soil P availability, a P concentration below the lower threshold of 1.1 mg/g DW may indicate that either the P nutritional status of the plant or the soil P availability or both are low. The same interpretations seem to apply to N/P ratios which, according to Mellert and Göttlein (2012) were in the normal range for BBR plants growing in BBR soil and above in all other cases.

### P Resorption From Senescent Leaves Is the First Reaction to Current Soil Conditions

Phosphorus resorption from senescent leaves is a nutrient conservation mechanism, common for several plant species including beech, which reduces losses, and decreases the nutrient uptake demand for the next year (Aerts, 1996; Brant and Chen, 2015). As a consequence, there is less input of P with litter fall diminishing a recycling pool in the ecosystem (Lang et al., 2017). The higher P resorption efficiency of plants grown in the LUE soil than for those grown in the BBR soil, irrespective of their P nutritional status, are in general agreement with findings from recent chronosequence studies by Richardson et al. (2004) and Hayes et al. (2014). The sensitive reaction of this parameter to the current soil in the first growing season, when the concentrations in mature green leaves still reflected the nutrient situation at the site of plant origin, confirms the findings of Hofmann et al. (2016) who showed that fertilization of beech saplings growing in soil low in P led to a decrease in P resorption efficiency. By contrast, the similar values of N resorption efficiency, measured in our experiment for all treatments, indicate a similar degree of N availability. The observed level of about 50% falls within the average range compiled by Aerts (1996) for deciduous trees and shrubs.

### P Allocation to Leaves Is a Sensitive Indicator of Soil P Availability

In this section we discuss in more detail the patterns of relative P and mass allocation to various plant compartments in the second growing season. While the link between nutrient allocation and a number of site factors such as precipitation, forest type, latitude, and plant age has been well studied (Sardans and Peñuelas, 2013), little is known about how soil P availability affects P allocation (Yang et al., 2016).

Let us first compare the saplings from LUE and BBR when growing in the soil from their site of origin. The lower allocation of mass and P to leaves and fine roots and higher allocation to the stem for the beech saplings from LUE are consistent with a conservative strategy reducing growth under nutrient limitation. Yang et al. (2016) also observed a slower growth of beech saplings from LUE than from BBR when growing in undisturbed soil cores from their own site of origin which represents a more natural situation than our experiment. A smaller root system in soil of low P availability is in line with the resource economics hypothesis (Grime, 1977; Craine, 2009) and the general characteristic that plant ecotypes from nutrient-limited environments grow slower than ecotypes from fertile soils. At the same time a tendency to a higher ratio between allocation of biomass to fine roots and leaves for the LUE saplings (mean ± SE: 2.0 ± 0.5) than for BBR saplings (mean ± SE: 1.6 ± 0.2) is in accordance with the notion that under nutrient-limited conditions plants should allocate proportionally more resources to roots (George et al., 2011). We do not know to what degree the difference in the size of the root system between these two treatments translates into volume of soil explored. In both treatments about 50% of the root tips were mycorrhized (mean ± SE: BBR in BBR, 49 ± 3%; LUE in LUE, 51 ± 10%), but extension of the hyphal system was not assessed.

When considering the two treatments with beech saplings growing in soil from the other site, the most striking result is that P allocation to leaves was the same as for the saplings adapted to the respective site, while mass allocation was intermediate in both cases. This indicates that P allocation to leaves might be a particularly good indicator of soil P availability, irrespective of P nutritional status of the plant. It further may be a key trait that the plant adjusts by balancing leaf biomass production against the transfer of nutrients from the soil or from/to internal storage organs such as stem or coarse roots.

Our results for the BBR plants growing in LUE soil indicate that the response of beech saplings with a high P nutritional status to low soil P availability is to decrease leaf P concentration, but to still produce as much leaf biomass as possible under the new low P flux into the roots. At this stage of plastic response, the substantial amount of P stored in coarse roots, and stem allows to partly compensate for the low P uptake. This compensation is also indicated by intermediate P concentrations in bark and wood exudates, maintaining the sink strength of leaves and roots at a low value similar to BBR plants growing in their own soil.

From the results for the LUE plants growing in BBR soil it appears that a beech sapling with low P nutritional status in response to high soil P availability increases leaf biomass production as much as possible under the new high P influx, while it keeps P concentrations in leaves low. Phosphorus concentrations in fine roots as high as for the saplings from BBR adapted to this soil, and the highest P allocation to fine roots of all treatments might be explained by inefficient P translocation to the aboveground plant compartments or by P recycling from stem and old leaves to support fine root growth (Marschner et al., 1996; Netzer et al., 2017). The higher sink strength of fine roots for LUE than BBR plants when growing in BBR soil argue rather in favor of inefficient translocation.

The significant difference in P allocation to leaves between beech saplings growing in BBR and LUE soil was not found for saplings growing in undisturbed soil cores from their own site (Zavišić and Polle, 2018; Zavišić et al., 2018). For a sampling time in July/August P allocation in saplings from both sites was similar to the one we measured for saplings growing in BBR soil, but also varied strongly during the growing season. This discrepancy may be explained by the presence of the organic surface layer in the undisturbed soil cores which could have increased the effective availability of soil P in the case of the LUE plants. The importance of the organic surface layer for the nutrition of young beech trees from soil low in P was clearly demonstrated by Hauenstein et al. (2018) who showed that the presence of the organic surface layer improved P nutrition and growth of beech seedlings in the LUE soil but had no effect in the BBR soil. The effect of the organic surface layer at LUE was attributed on one hand to a particularly high microbial activity promoted by a high root density, but also to a high water retention capacity minimizing loss of added or mobilized P to the mineral soil (Hauenstein et al., 2018). In particular, the high water holding capacity of the surface layer at LUE might have led to a similar effective P availability in the LUE soil as in the BBR soil during the well watered experiments with undisturbed soil cores. On the other hand, at on average drier conditions in the field one would expect a smaller effect, being consistent with the lower P nutritional status of beech saplings from LUE than from BBR.

Furthermore, we cannot exclude that stress induced by the transplantation process (accidental cutting of some roots during sampling, dying-off of fine roots during storage between sampling, and planting) may have affected P allocation even in the second growing season. In particular, “recovery” of a mycorrhized root system in the new soil could have led to a temporary elevated biomass and P allocation to the fine roots. A comparison with the more natural situation in the studies of Zavišić and Polle (2018) and Zavišić et al. (2018) revealed that biomass and P allocation were twice as high in our rhizoboxes than in the undisturbed soil columns of the mentioned studies for saplings from BBR but similar for saplings from LUE. Considering (i) the high variability among individual saplings, (ii) the differences in environmental conditions between the rhizobox and column studies, and (iii) that the larger differences occurred for the more fertile soil from BBR, this comparison does not indicate a large effect of transplantation in our rhizoboxes.

### Phosphorus Allocation to Leaves and P Concentrations in Fine Roots Represent Best the Plastic Response of Beech to Changes in Soil P Availability

In this section we discuss the differences in various plant traits among the experimental treatments in terms of a plastic response of beech saplings to changes in soil P availability. For this, we assume that the trait values of saplings growing in the soil from their site of origin represent the extremes and thus define the potential trait span. We further assume, that the higher the degree to which the value of this trait changes relative to the trait span for a sapling exposed to soil from the other site, the higher is the plastic response of this trait. We did neither include P allocation to fine roots nor P and mass allocation to coarse roots, because there was no significant trait span according to the definition. We interpret the differences between the trait plasticity for BBR plants exposed to low P soil and for LUE plants exposed to high P soil as differences between the acclimation processes from high to low and from low to high soil P availability.

Considering all our plasticity indices, they indicate a rather high plasticity compared with other studies employing various plasticity quantification methods (Valladares et al., 2002; Stojnić et al., 2013, 2015a). The direction of acclimation affected the plasticity index for some traits but was unimportant for others. While the plasticity of P resorption and P concentration in leaves was higher for the beech saplings with a high P nutritional status acclimating to low P soil, P and mass allocation to the stem were more reactive for saplings with a low P nutritional status acclimating to soil with high P availability. The asymmetry in size of the reaction of the mentioned traits emphasizes the importance of taking into account the initial nutritional status of a plant when assessing plasticity. On the other hand, the strong responses of P allocation to leaves and P concentration in fine roots, which were similar for both directions of acclimation, suggest that these plant traits are most suitable to assess plasticity of beech in response to soil P availability.

## Conclusion

A two-year cross-growth experiment with beech saplings and mineral soil from two forest sites differing strongly in soil P availability, demonstrated a high plasticity of juvenile *F. sylvatica* to differences in soil P availability. Some influence of recovery from stress implicated by the transplantation on these results, can however, not be excluded. Relative P allocation to leaves appears to be a particularly good indicator of soil P availability, irrespective of the P nutritional status of the plant.

In contrast to P allocation to leaves, foliar P concentrations were not a clear indicator of soil P availability, which may partly explain the lack of relation between leaf P concentrations and soil P availability in studies comparing forest sites on the European or regional scale (Talkner et al., 2015; Lang et al., 2017). In particular, the ambiguity with respect to the interpretation of foliar P concentrations below threshold values for normal growth implies that the observation of such low values for beech on monitoring sites (Jonard et al., 2015; Talkner et al., 2015) not necessarily indicates P deficiency and respective growth reduction, but rather a tree with still good P nutritional status but reacting to a decrease in soil P availability.

Overall, the results clearly rebut our working hypothesis, that adaptation of beech saplings to new soil conditions is driven by the plant striving to achieve or maintain high foliar P concentrations. By contrast, they point to a sensitive signaling network that allows the plant to produce as much biomass as possible under given soil conditions by regulating mass and nutrient allocation accordingly.

## Data Availability

The datasets generated for this study are available on request to the corresponding author.

## Author Contributions

All authors designed the experiment and contributed significantly to the final version of the manuscript. SM and JL collected the plant and soil materials used in the study. SM set-up and carried out the experiment, performed most of the chemical analyses, analyzed the data, and wrote the first version of the manuscript.

## Conflict of Interest Statement

The authors declare that the research was conducted in the absence of any commercial or financial relationships that could be construed as a potential conflict of interest.

## Footnotes

1 http://www.ecosystem-nutrition.uni-freiburg.de/
